# Metabolic and physiological responses to progressive drought stress in bread wheat

**DOI:** 10.1038/s41598-020-74303-6

**Published:** 2020-10-14

**Authors:** Michael Itam, Ryosuke Mega, Shota Tadano, Mostafa Abdelrahman, Sachiko Matsunaga, Yuji Yamasaki, Kinya Akashi, Hisashi Tsujimoto

**Affiliations:** 1 United Graduate School of Agricultural Sciences, Tottori University, Tottori, 680-8553 Japan; 2 Arid Land Research Center, Tottori University, Tottori, 6800001 Japan; 3 Graduate School of Sciences and Technology for Innovation, Yamaguchi University, Yamaguchi, 753-8515 Japan; 4 Botany Department, Faculty of Science, Aswan University, Aswan, 81528 Egypt; 5 Faculty of Agriculture, Tottori University, Tottori, 680-8553 Japan

**Keywords:** Plant sciences, Plant stress responses, Drought

## Abstract

Wheat (*Tritium aestivum*) is vulnerable to future climate change because it is predominantly grown under rain-fed conditions in drought-prone areas. Thus, in-depth understanding of drought effect on wheat metabolism is essential for developing drought-tolerant wheat varieties. Here, we exposed wheat ‘Norin 61’ plants to progressive drought stress [0 (before drought), 2, 4, 6, 8, and 10 days after withholding water] during the flowering stage to investigate physiological and metabolomic responses. Transcriptional analyses of key abscisic acid-responsive genes indicated that abscisic acid signalling played a major role in the adaptation of wheat to water deficit. Carbon isotope composition had a higher value than the control while canopy temperature (CT) increased under drought stress. The CT depression was tightly correlated with soil water potential (SWP). Additionally, SWP at − 517 kPa was identified as the critical point for increasing CT and inducing reactive oxygen species. Metabolome analysis identified four potential drought-responsive biomarkers, the enhancement of nitrogen recycling through purine and pyrimidine metabolism, drought-induced senescence based on 1-aminocyclopropane-1-carboxylic acid and Asn accumulation, and an anti-senescence response through serotonin accumulation under severe drought stress. Our findings provide in-depth insight into molecular, physiological and metabolite changes involved in drought response which are useful for wheat breeding programs to develop drought-tolerant wheat varieties.

## Introduction

Wheat (*Triticum aestivum*) is one of the most important staple-food crops and key sources of food calories especially to the ~ 4.5 billion people living in developing countries^1^. However, wheat yield is estimated to be reduced by ~ 6.0% per °C rise in global mean temperature, in concomitance with frequent exposures to prolonged drought episodes as a result of climate change^2,3^. Water availability is crucial for wheat production, and thus drought stress is considered a major factor affecting wheat yield losses^4^. With predicted increase in world population to 9.6 billion by 2050^5^ agricultural water supply must be increased by ~ 17% to maintain agricultural productivity^6^. In addition, wheat demand is increasing in developing countries, and consumption rate in sub-Saharan Africa recently reached ~ 650 million tons per year^7^, causing additional pressure on wheat demand. Thus, the generation of drought-tolerant wheat varieties with greater water-use efficiency is of the utmost priority, especially in the context of food sustainability. However, this requires a detailed understanding of wheat physiological and metabolic responses to drought stress.

Abscisic acid (ABA) biosynthesis is induced when plants respond to drought stress, and subsequently increased ABA binds to its receptor to initiate signal transduction, leading to stomatal closure and other cellular responses to stress^8^. By closing the stomata, transpiration is suppressed and plants are able to prevent water loss and maintain sufficient level of water under drought condition. A recent report on wheat drought tolerance indicates that wheat plants overexpressing ABA receptor (*TaPYL4*) improved seed production per L of supplied water in comparison with wild-type plants^9^. Drought-responsive metabolites such as Pro were induced by ABA^10,11^, and the rate-limiting gene for Pro biosynthesis, *Δ1-pyrroline-5-carboxylate synthase* (*P5CS*), is also controlled by the ABA-signalling pathway^12^.

Metabolomics has become a powerful tool in the post-genomics era, enabling us to explore different aspects of the biological and physiological changes caused by environmental or genetic perturbations^3^. In addition, metabolites come last in the omics cascade (that is, relatively close to the phenotype) and are therefore, a reliable tool for investigating abiotic stress responses in plants^13^. For example, branched chain amino acids (BCAAs), respiratory amino acids (Gly and Ser), and some of the tricarboxylic acid cycle-intermediates are known to be accumulated in *Arabidopsis thaliana*, rice (*Oryza sativa*), and barley (*Hordeum vulgare*) in response to drought stress^14–16^. In wheat, accumulations of Pro, Trp, organic acids, phenolics, and sulphur-related metabolites (glutathione, Met, and Cys) have been reported under drought-stress conditions^17–20^. However, there is limited knowledge on specific soil moisture conditions associated with particular metabolic profiles in wheat. To advance this knowledge, it is necessary to conduct a time-lapse study having many sampling points under progressive drought-stress conditions.

The objective of this study was to elucidate the physiological and metabolic responses of wheat ‘Norin 61’ to progressive drought stress at the flowering stage, as a critical stage affecting grain yield^21^. We subjected wheat plants to progressive drought stress in a growth chamber by monitoring the conditions for 10 days. Then, we analysed the relationship between drought level based on soil water potential (SWP) and physiological responses such as canopy temperature (CT), reactive oxygen species (ROS), and carbon isotope composition (δ^13^C). In parallel, we analysed metabolite changes and ABA-responsive gene expression under respective time points. Our findings (i) demonstrated a strong association between canopy temperature depression (CTD) and SWP, (ii) identified a threshold moisture content triggering maximum plant response, and (iii) drought response-related metabolite biomarkers. Our findings provide comprehensive information on physiological and metabolic dynamics associated with drought stress tolerance in wheat, which would be a major step for accelerating the development of wheat-tolerant varieties using biomarker-assisted selection.

## Results

### Physiological effects of progressive drought stress in wheat

**Figure 1:**
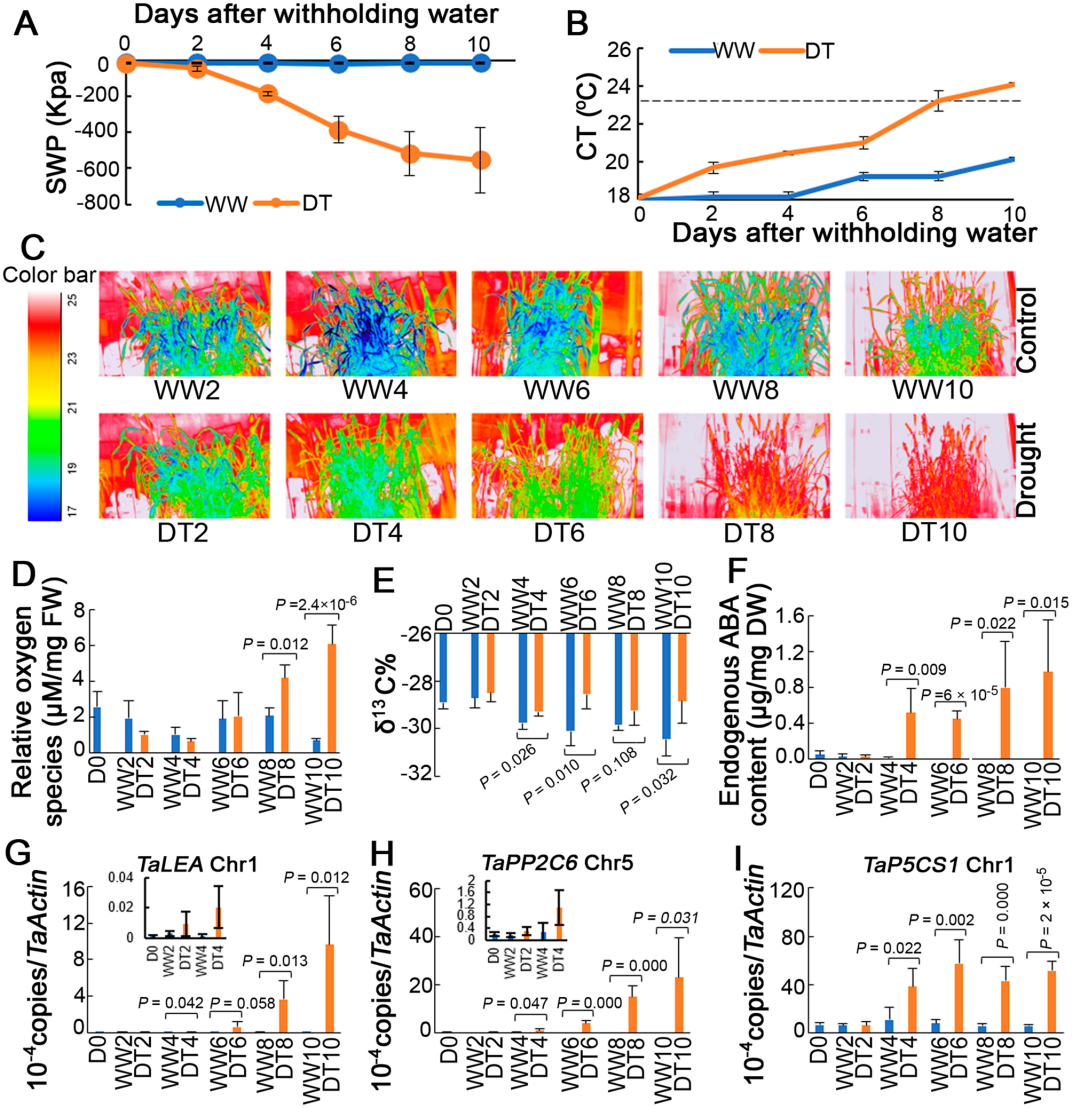
Physiological changes in wheat ‘Norin 61’ under progressive drought stress. (**A**) Decreasing soil water potential (SWP) under 10 days of progressive drought stress. Data represent mean ± standard deviation (SD) of four replicates. (**B**) Canopy temperature (CT) of plants under drought (DT) and well-watered (WW) conditions. Data represent mean ± SD of three biological replicates (one replicate consisted of three plants per pot) with 20 data points from each pot. Dashed line represents ambient temperature. (**C**) Thermal images of plants under different time points of DT and WW conditions. (**D**) Increase in reactive oxygen species in flag leaves of plants under DT in comparison with WW using spectrophotometry at 480 nm (excitation)/530 nm (detection). (**E**) Increase in carbon isotope composition (δ^13^C) in flag leaves of plants under DT compared with WW. (**F**) Increase in abscisic acid (ABA) concentration in flag leaves of plants under DT and WW conditions. Endogenous ABA contents were measured using LC–MS and calculated with ABA standard mixtures of different concentrations (0, 0.4, 2 and 10 ppm). (**G**–**I**) Relative expression of ABA-responsive genes in response to drought stress. Data represent mean ± SD of four biological replicates. Numbers represent days after withholding water. Inset bar graphs indicate each figure magnified in early treatment regions.

To evaluate drought-stress levels, the SWC was monitored using sensors, and the values were converted to SWP to ensure replicability in different soils. The SWP was maintained at − 15.8 kPa in control pots but decreased continuously in drought-treated pots as the drought intensified. The steady decline in SWP ensured that plants were subjected to progressive drought stress. The SWP reduction rate was high during the early days of drought treatment (DT2, 4, and 6), decreasing from − 45.1 kPa on DT2 to − 385.1 kPa on DT6 (Fig. 1A and Table 1), but was low during day 8 and 10 (DT8 and 10, respectively), decreasing from − 517.7 kPa on DT8 to − 554.5 kPa on DT10 as the drought intensified.

**Table 1:** Comparisons of canopy temperature depressions (CTDs) and soil water potentials (SWPs).

Treatment days	SWP in WW (kPa)	CTD in WW (°C)	SWP in DT (kPa)	CTD in DT (°C)
0	− 15.8	5.48	− 17.0	5.38
2	− 14.5	5.33	− 45.1	3.77
4	− 15.5	5.32	− 185.0	2.99
6	− 16.4	4.22	− 385.1	2.48
8	− 15.8	4.22	− 517.7	0.25
10	− 14.8	3.34	− 554.7	-0.62

A strong positive correlation (r^2^ = 0.95, *P* < 0.05) exists between CTD and SWP under drought-stress conditions.

*DT* drought-treatment, *WW* well-watered control. CTD data represent means of three pots. SWP data represent means of four pots.

To understand the CT changes in response to different drought levels, the thermal analysis of plants was performed under progressive drought stress as shown in Fig. 1B. Prior to drought treatment (D0), the CTs of both drought-treated and well-watered plants were similar, and the CTDs were ~ 5 °C (Table 1), indicating that plants were not under stress^22^. However, during DT2, drought-treated plants began to show slight increases in CT (Fig. 1B,C), and the CTDs were 5.3 °C and 3.7 °C for well-watered and drought-treated plants, respectively. The CT increased continuously, while the corresponding CTD decreased as the drought intensified. A sharp increase in CT was observed during DT8 as the SWP decreased to − 517.7 kPa (Fig. 1A,C and Table 1). Overall, the greatest increases in CT were observed during DT8 and 10, with CTDs of 0.25 °C and − 0.62 °C, respectively, while the corresponding well-watered control (WW8 and 10, respectively) had CTDs of 4.22 °C and 3.34 °C, respectively (Table 1). Consequently, there was a strong positive correlation between CTD and SWP (r^2^ = 0.95, *P* < 0.05). This suggests that DT8 is a physiologically critical point. Using these soil and plant states, we analysed ROS, carbon isotope composition, metabolite changes, and ABA-responsive gene expression levels to better understand plant responses to progressive drought stress.

### Gradual ROS accumulation under progressive drought stress

To investigate the oxidative stress effects of progressive drought stress, the ROS content was measured using flag leaf samples collected at each progressive drought point. The ROS contents of drought-treated samples increased significantly on DT8 and 10 (Fig. 1D). This suggests that at DT8 and 10, the ROS generation rate exceeded the plants’ scavenging capability owing to severe drought stress. However, there were no significant changes during DT2, 4, and 6, indicating that there was no severe drought stress at these time points.

### ^13^C composition under progressive drought stress

Drought causes stomatal closure which affects photosynthetic carbon isotope discrimination^23^. To evaluate the stress levels in plants under progressive drought conditions, the ^13^C composition in flag leaf samples was investigated. The ^13^C composition in drought-treated samples had higher values than the control conditions starting from DT4 (Fig. 1E). At DT8, the δ^13^C value was slightly higher but not significantly different from the control. Overall, this indicates that there was an alteration in carbon isotope discrimination in response to progressive drought stress.

### ABA-responsive gene expression under progressive drought stress

ABA is well-known to be biosynthesized in response to drought stress. Endogenous ABA contents and ABA responsive genes are often utilized to confirm the effect of drought treatment. Therefore, we investigated the transcript levels of ABA-responsive genes and endogenous ABA level with qRT-PCR and LC–MS, respectively. At first, LC–MS measurements indicated that ABA accumulated significantly from DT4 to 10, suggesting that ABA biosynthesis occurred starting from DT4 (Fig. 1F). Two ABA-responsive genes: the late embryogenesis abundance gene, *TaLEA* and the ABA-signalling negative regulator gene, *TaPP2C6* were previously reported in wheat under drought stress^9^. Also, the Pro biosynthetic gene, *P5CS* was proposed as an ABA responsive gene in Arabidopsis^12^. The expression levels of *TaLEA*, *TaPP2C6*, and *TaP5CS* significantly increased under drought-stress conditions, starting from DT4, indicating that these genes were upregulated in response to drought-induced ABA accumulation (Fig. 1G–I). This suggests that DT4 is the start point of ABA response.

### Total metabolite profiling under progressive drought stress

**Figure 2:**
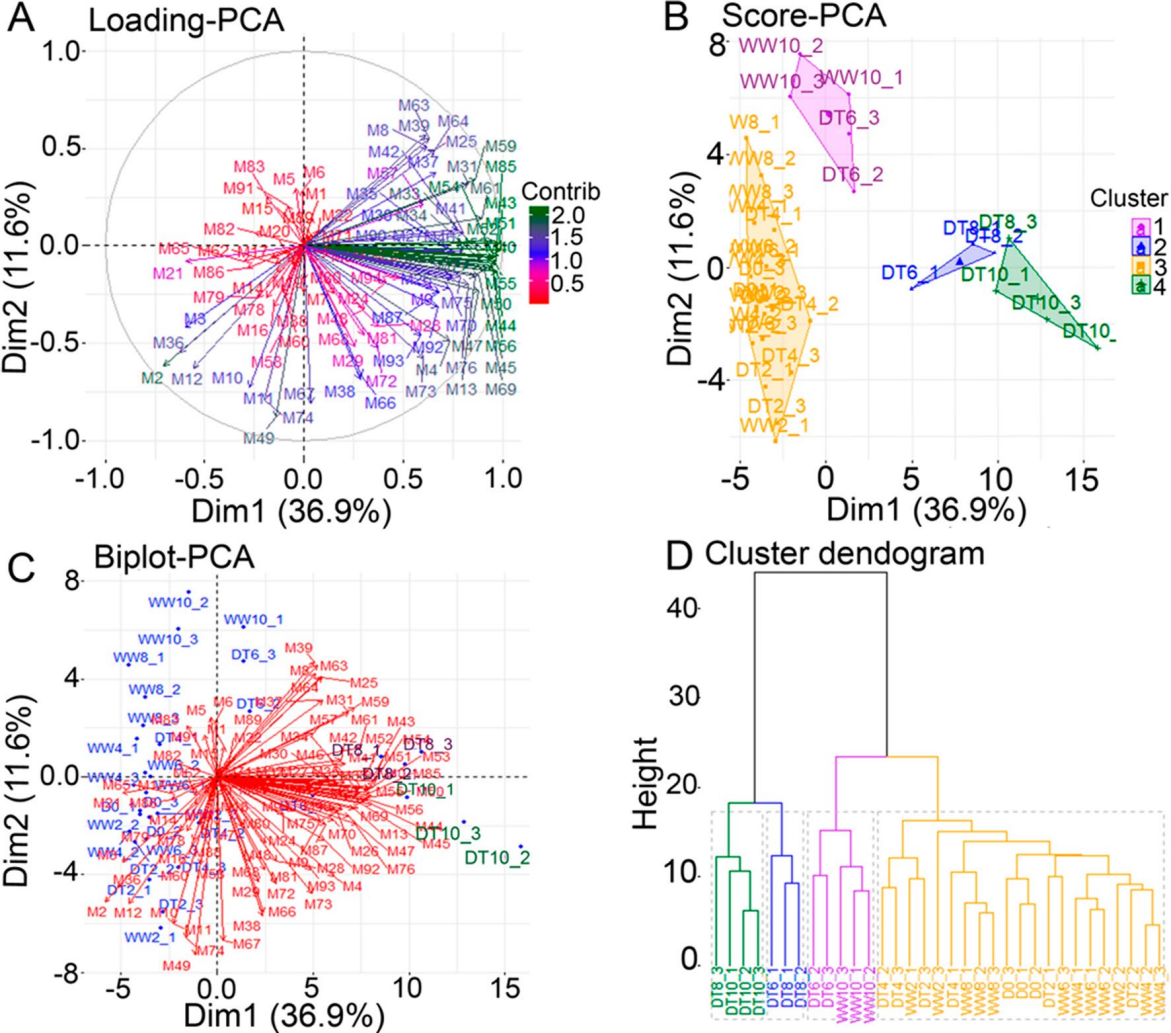
Principal component analysis (PCA) and dendrogram clustering of the interrelated effects of progressive drought (DT) stress and well-watered (WW) conditions on the metabolic profile of wheat ‘Norin 61’. (**A**) PCA loading plot of the metabolite (M) variables identified in wheat ‘Norin 61’ under different time points of drought (DT2, 4, 6, 8 and 10) and well-watered (WW2, 4, 6, 8 and 10) conditions. Metabolites with high contribution to PC1 and PC2 axes are shown in green. (**B**) PCA score plot of flag leaf samples collected from different DT and WW conditions according to their metabolite profiles. (**C**) PCA Biplot showing combined metabolite and condition trends (**D**) K-means cluster dendrogram further confirming the clustering of flag leaf samples based on metabolite profiles. DT10 and DT8 are clearly separated from well-watered samples, indicating severe drought stress. Data represent three biological replicates. Numbers following DT or WW represent days after treatment, while numbers following the underbars represent individual replicate numbers.

To evaluate the metabolite changes involved in progressive drought responses, 94 metabolites were quantified using flag leaves of plants grown under well-watered and drought conditions. Subjecting the metabolite data to a PCA revealed the high variability in metabolite contributions to drought responses (Fig. 2A). PC1 (Dim 1) explained 36.9%, while PC2 (Dim 2) explained 11.6% of the variability. More than one-half of all metabolites, especially amino acids, nucleosides, and organic acids were affected by severe drought stress at DT8 and 10 (Fig. 2A–C and Supplementary Table S2). All the well-watered samples (D0, WW2, 4, 6, 8, and 10) tended to cluster together, indicating that they had similar metabolite profiles. In addition, samples grown under mild drought conditions (DT2 and 4) clustered towards the well-watered samples (Fig. 2B,D). Two out of three samples at DT6 were located in the intermediate region between mild and severe drought (Fig. 2B,D), suggesting that DT6 is a transition state.

### Metabolite changes at each drought level

**Figure 3:**
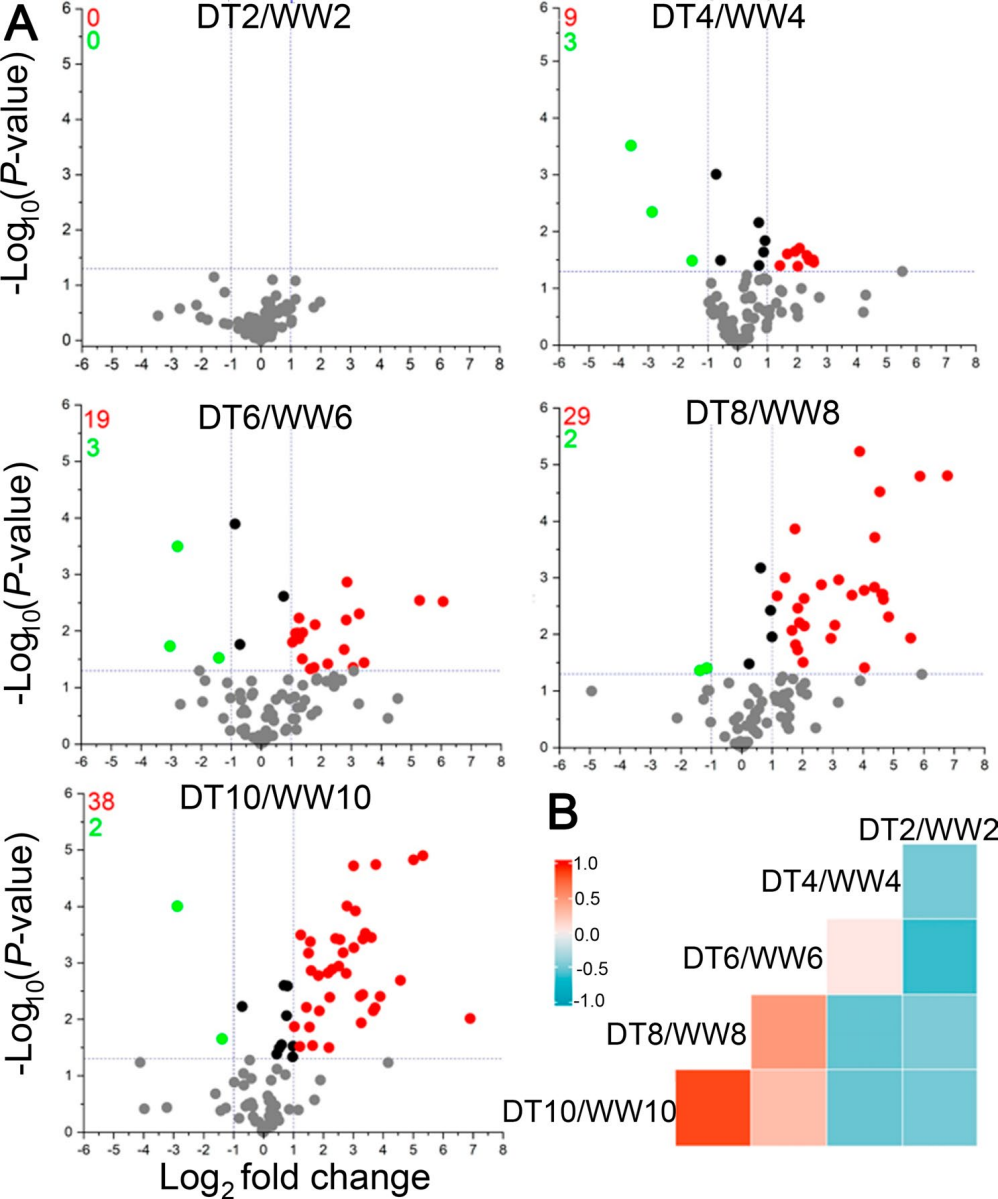
Metabolite trends in wheat ‘Norin 61’ under different drought levels (DT2, 4, 6, 8, and 10, respectively) relative to well-watered (WW) conditions. (**A**) Volcano plots of differentially accumulated metabolites. The threshold of significantly (*P* < 0.05) downregulated (green dots, fold change ≤ 0.5) and upregulated metabolites (red dots, fold change ≥ 2.0) are highlighted. (**B**) Treatment-treatment correlations in ‘Norin 61’ in response to DT versus WW at different time points.

Then, significantly up- and down-regulated metabolites (> twofold change, *P* < 0.05) were characterized by volcano plots under each drought condition. At DT2, there was no significant metabolite change, but starting from DT4, the number of upregulated metabolites increased along with drought intensity (Fig. 3A). In contrast, the numbers of downregulated metabolites were similar in a drought level-dependent manner. At DT4, DT6, DT8, and DT10, 9, 19, 28, and 38 metabolites, respectively, were upregulated, while 3, 3, 2, and 2 metabolites, respectively, were downregulated. In addition, correlation analyses among drought conditions indicated positive correlations (*r* = 0.66 and 0.51) among severe drought stress time points, DT8/WW8–DT10/WW10 and DT6/WW6–DT8/WW8, respectively, while low correlations (*r* = 0.25 and 0.20 ) were indentified among severe and mild drought stress time points, DT4/WW4–DT6/WW6 and DT4/WW4–DT10/WW10, respectively (Fig. 3B). The correlations between the DT2 samples and the other samples were less than 0.2. This correlation analysis suggested that the drought levels from DT6 to 10 were more similar than those of DT2 to 4. This finding also indicated that drought stress rapidly increased between DT4 and 6, and, therefore, DT6 is likely a transition stage.

**Figure 4:**
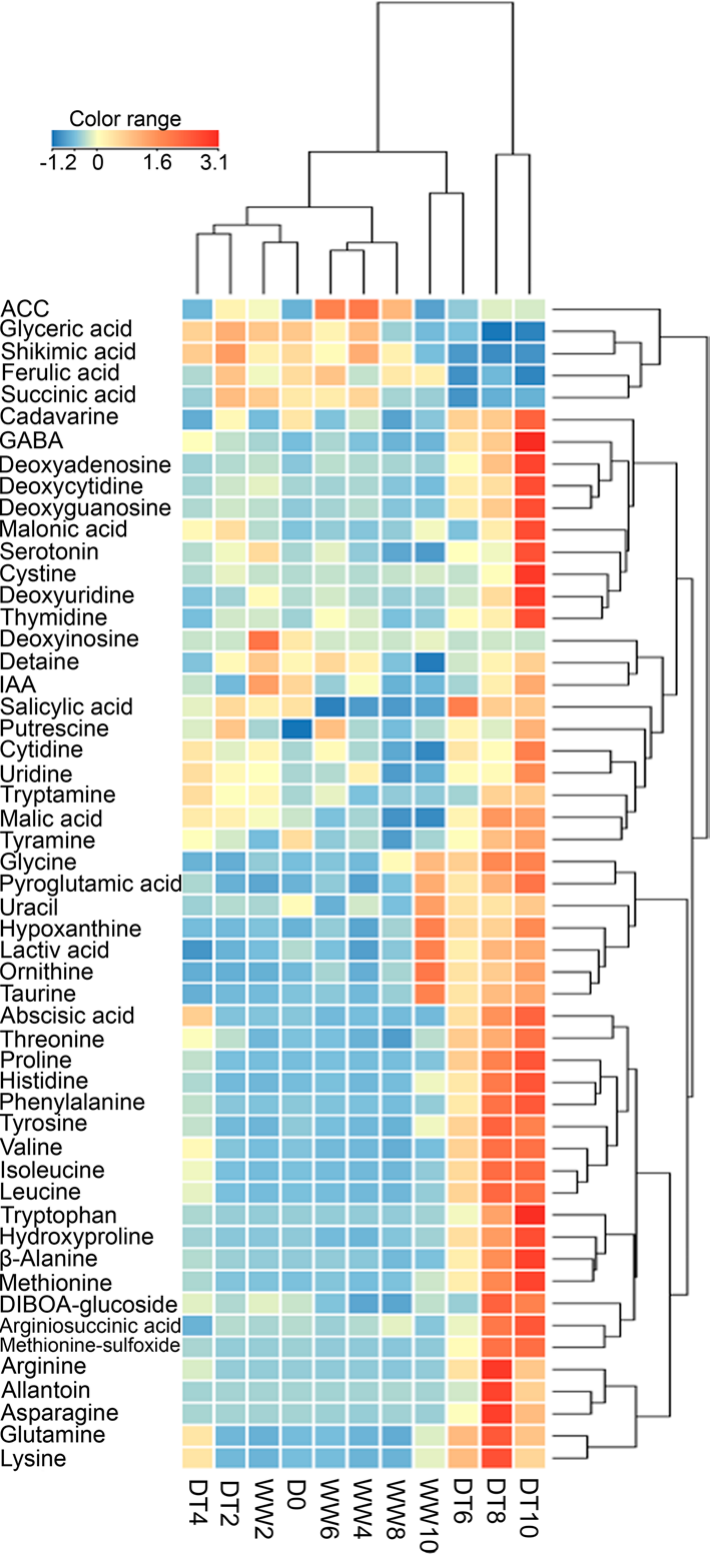
Hierarchical clustering of metabolite levels of ‘Norin 61’ in response to drought (DT) and well-watered (WW) conditions. The Z-score transformation of the mean values of 53 significantly (*P* ≤ 0.05) increased (fold change ≥ 2.0) or decreased (fold change ≤ 0.5) metabolite intensities were used for hierarchical clustering. Data represent three independent biological replicates in each condition. Red fields indicate high accumulation rates, yellow and blue fields indicate low and very low accumulation rates, respectively.

Among the 94 metabolites analysed, the 53 metabolites with the greatest accumulations (≥ twofold change, *P* < 0.05) were selected for further analyses. A hierarchical clustering analysis of the 53 metabolites showed an overview of their accumulation levels under different drought conditions (Fig. 4), with DT6 clustering between severe drought and control categories, which further indicated that DT6 is a transition state in the progressive drought responses.

**Figure 5:**
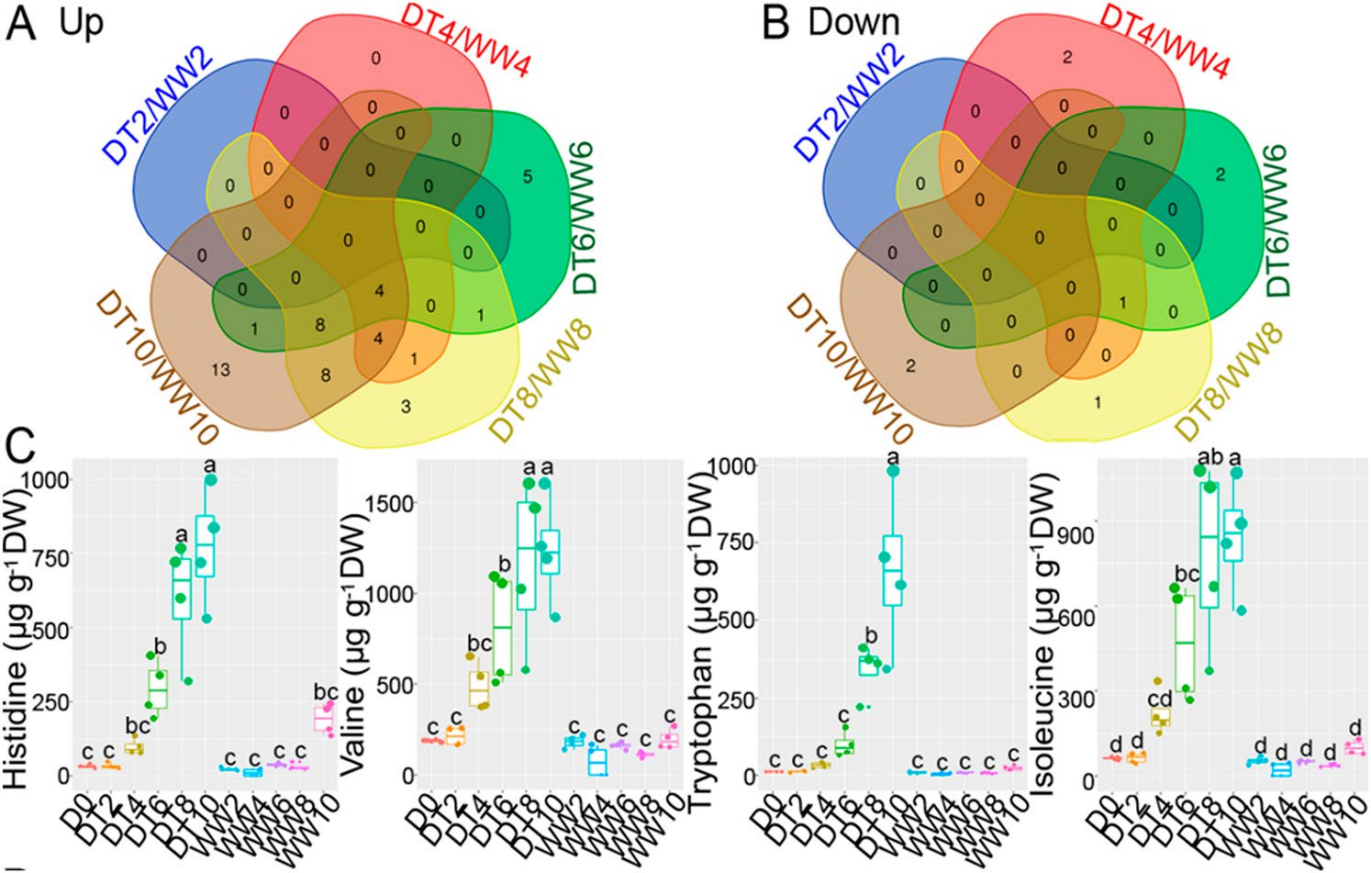
Metabolite dynamics in wheat ‘Norin 61’ after 2, 4, 6, 8, and 10 days exposure to drought stress (DT2, 4, 6, 8, and 10, respectively) relative to well-watered (WW) conditions. (**A**,**B**) Venn diagram of unique and overlapped significantly increased (*P* ≤ 0.05, fold change ≥ 2.0) and decreased (*P* ≤ 0.05, fold change ≤ 0.5) metabolites in response to drought stress. (**C**) Box plot analysis of four commonly increased metabolites at DT4, 6, 8, and 10 relative to WW conditions. Different letters indicate significant difference (*P* < 0.001) according to Tukey’s honesty significant difference (HSD) test.

Venn diagrams were used to reveal the condition-related specificities of the metabolites (Fig. 5). Five metabolites (Gly, taurine, hypoxanthine, lactic acid, and ornithine) were specifically upregulated at DT6, 3 (uracil, tyramine, and methionine-sulfoxide) at DT8, and 13 (malonic acid, putrescine, thymidine, uridine, Cys-Cys, deoxyuridine, betaine, serotonin, cytidine, 1-aminocyclopropane-1-carboxylic acid (ACC), tryptamine, indole-3-acetic acid, and allantoin) at DT10 (Fig. 5A, Supplementary Table S3). Among the DT10-specific metabolites, allantoin (119-fold), ACC (13-fold), and tryptamine (12-fold) had the highest fold changes (Table 2 and Supplementary Table S2). Similarly, some metabolites were upregulated at two different drought levels. For example, pyroglutamic acid was upregulated at DT6 and 8. Eight metabolites [2,4-dihydroxy-1,4-benzoxazin-3-one-glucoside, Asn, cadavarine, gamma-aminobutyric acid (GABA), malic acid, deoxyadenosine, arginosuccinic acid, and Tyr] were upregulated at DT8 and 10 (Table 2). Pyruvic acid and arginosuccinic acid were downregulated at DT4, succinic acid and shikimic acid at DT6, glyceric acid and ACC at DT8, and deoxyinosine and ferulic acid at DT10 (Fig. 5B, Supplementary Table S3). Interestingly, four metabolites (His, Val, Trp, and Ile) were consistently upregulated from DT4 to 10 (Fig. 5C and Supplementary Table S2). These metabolites are probably drought-responsive and may be used as potential biomarkers.

**Table 2:** Metabolites specifically upregulated under severe drought conditions on days 8 and 10 (DT8 and 10, respectively).

Metabolites	Label	Condition	Fold change	*P*-value
Uracil	M57	DT8	3.5	0.0189
Tyramine	M92	DT8	2.2	0.0020
Met-Sul	M34	DT8	28.6	0.0049
DIBOA-Glc	M13	DT8, DT10	6.1, 2.9	0.0013, 0.0004
Asn	M27	DT8, DT10	16.5, 9.6	0.0389, 0.0115
Cadaverine	M90	DT8, DT10	4.1, 3.5	0.0023, 0.0070
GABA	M47	DT8, DT10	3.3, 6.9	0.0001, 0.0000
Malic acid	M4	DT8, DT10	2.6, 2.3	0.0009, 0.0003
Deoxyadenosine	M77	DT8, DT10	3.6, 5.3	0.0034, 0.0003
Arg-Suc	M45	DT8, DT10	3.7, 9.9	0.0062, 0.0036
Tyr	M52	DT8, DT10	9.1, 2.8	0.0010, 0.0136
Malonic acid	M9	DT10	4.5	0.0316
Putrescine	M48	DT10	2	0.0134
Thymidine	M70	DT10	3.5	0.0017
Uridine	M72	DT10	2.7	0.0061
Cys-Cys	M26	DT10	8.4	0.0001
Deoxyuridine	M75	DT10	6.3	0.0007
Betaine	M38	DT10	3.1	0.0292
Serotonin	M93	DT10	8	0.0000
Cytidine	M73	DT10	2.8	0.0007
ACC	M86	DT10	13.2	0.0062
Tryptamine	M94	DT10	12.7	0.0071
IAA	M80	DT10	4.6	0.0041
Allantoin	M30	DT10	119.5	0.0096

*DIBOA* 2,4-dihydroxy-1,4-benzoxazin-3-one, *GABA* gamma-aminobutyric acid, *ACC* 1-aminocyclopropane-1-carboxylic acid, *IAA* indole-3-acetic acid.

### Pathway analysis based on metabolite enrichment under progressive drought stress

**Figure 6:**
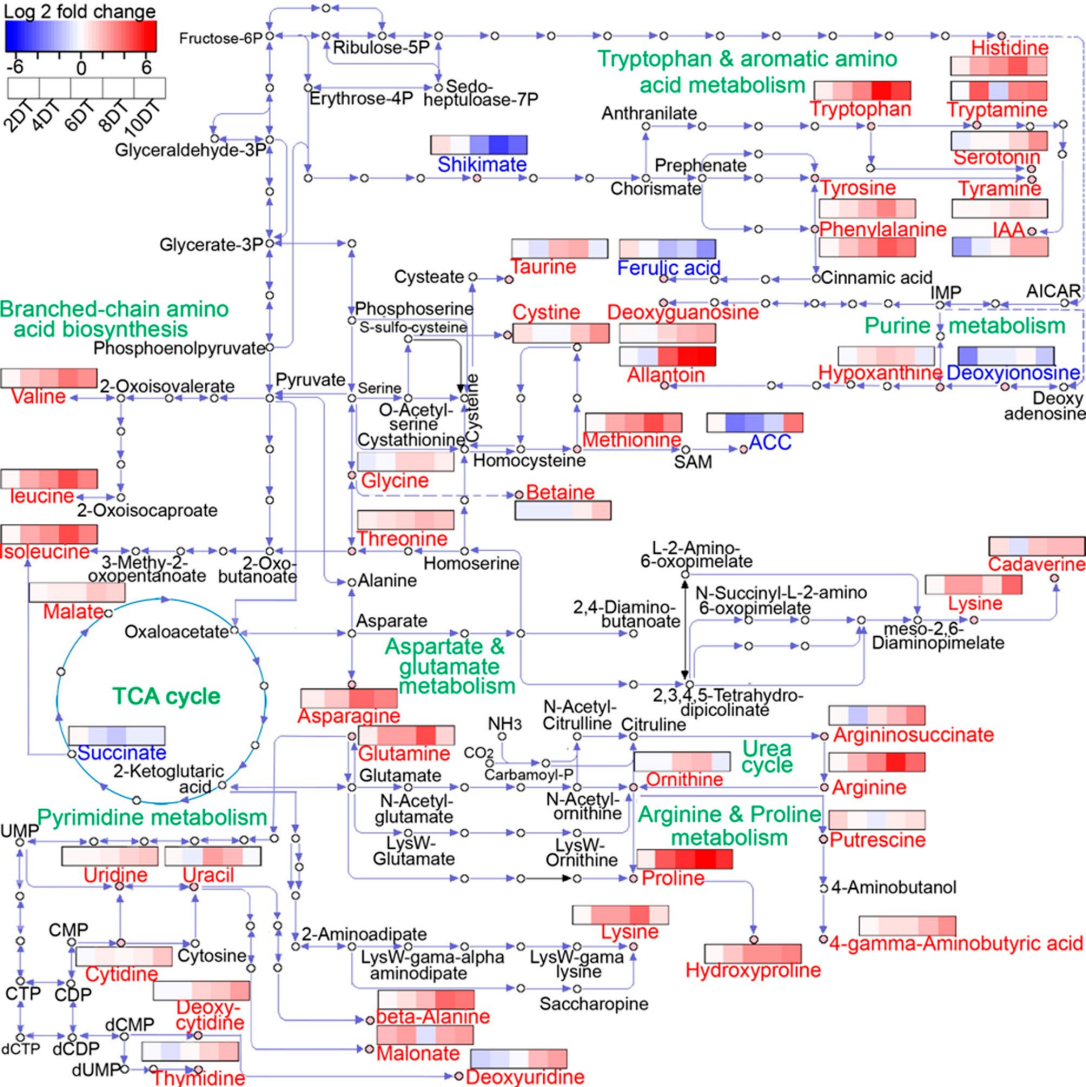
Metabolite changes in flag leaves of wheat ‘Norin 61’ during 10 days of progressive drought stress (DT2, 4, 6, 8 and 10). The proposed metabolic pathways are based on KEGG database (https://www.genome.jp/kegg/) and the literature. Metabolites with significant (*P* ≤ 0.05) increase or decrease (fold change > 2.0 or fold change ≤ 0.5, respectively) and are shown in the pathway. Color bar indicates the log_2_ fold change.

**Figure 7:**
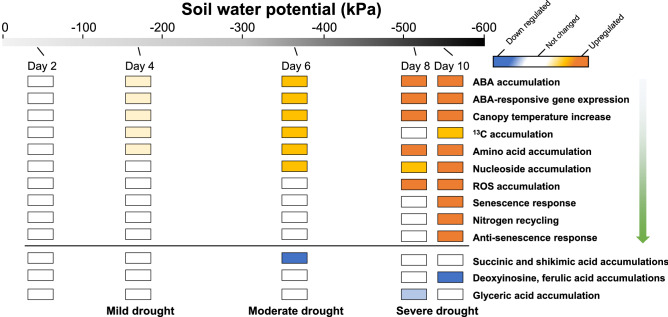
A schematic diagram of wheat ‘Norin 61’ response to progressive drought stress. Changes in physiological and metabolic traits were evaluated from experimental results. The arrow indicates the observed sequence of drought-responsive changes.

Finally, metabolite pathway analysis was conducted. Most metabolites showed increasing trends under drought-stress conditions, although shikimic, ferulic, and glyceric acids showed decreasing trends (Figs. 6 and 7). Taken together, the major metabolic pathways affected in this study, based on pathway and network analyses, include the following: (i) aspartate (ii) pyrimidine, (iii) glycine and serine, (iv) arginine and proline, (v) urea cycle (vi) tryptophan and aromatic amino acid, (vii) BCAA, and (viii) purine metabolism (Fig. 6, Supplementary Fig. S2).

## Discussion

CT is an important tool for studying plant physiological responses to drought stress, because it integrates many physiological responses into a single low-cost measurement^25^. In the present study, the CT of wheat increased, while the corresponding CTD decreased under progressive drought-stress conditions. The increase in CT supports previous reports in which drought caused stomatal closure, leading to increased respiration and reduced transpiration^26^. Our study indicates that the reduction in water consumption, especially during DT8 (− 517 kPa), is responsible for the sharp rise in CT (Fig. 1B,C) and suggests that although ABA accumulation began in DT4, drastic stomatal closure occured in DT8 in response to the drought stress (Fig. 1F). Consistently, increase in ABA-responsive gene (*TaLEA*, *TaPP2C6* and *TaP5CS*) expressions were observed under drought conditions starting from DT4, indicating that ABA signalling was earlier upregulated in response to the drought stress (Fig. 1F–I). This confirms that physiological response to drought occured after ABA biosynthesis. These results support the findings of Merlot et al.^27^ in which the use of CT values for the genetic dissection of ABA biosynthesis in Arabidopsis was validated. There was a strong correlation (r^2^ = 0.95, *P* < 0.05; Table 1) between CTD and SWP. The CTD explained 95% of the changes in SWP resulting from drought stress. This implies that a simple CT reading can accurately (*P* < 0.05) predict the SWP or drought-stress level in wheat without disturbing the plant.

^13^C composition analyses have been used to evaluate plant responses to drought stress^23^. In this study, the higher value than the control in the ^13^C composition of wheat at each point (Fig. 1E) suggested a change in the normal carbon isotope discrimination in response to drought stress. This indicated that stomatal closure was induced at various time points due to drought stress. Consequently, a continuous increase in CTs was observed alongside ABA-responsive gene expression levels, indicating that plants were subjected to progressive drought stress.

Abiotic stresses have profound effects on plant metabolism, and, as a result, metabolomics is a burgeoning research field. In this study, we used a metabolomics analysis to dissect the metabolite changes in wheat in response to different drought levels during a 10-d progressive drought period. The most pronounced changes were increases in amino acids, organic acids, and nucleosides. Four amino acids (His, Val, Trp, and Ile) were consistently and rapidly upregulated from DT4 to 10 (from mild to severe drought), suggesting that they are drought biomarkers (Fig. 5B). Amino acid accumulations, such as BCAAs and aromatic amino acids, in drought-stressed plants have overall beneficial effects on the stress acclimation^17,28,29^. Organic acids, such as lactic, malic, and succinic acids, increased in response to drought stress. Although the roles of organic acids in drought response and adaptation are not fully understood, they may accumulate owing to drought-induced perturbations of the tricarboxylic acid cycle^17,19,29^. Similarly, salicylic acid, a plant hormone, accumulated alongside ABA in response to drought stress starting from DT6 (Supplementary Table S2). Salicylic acid, like ABA, is involved in stomatal regulation through Ca^2+^-dependent protein kinases located downstream of the peroxidase-mediated ROS signalling pathway in Arabidopsis guard cells^30^. Thus, ABA and salicylic acid may co-regulate stomatal closure in response to drought stress.

Pro, a well-known marker for drought response, accumulated starting from DT6 (Fig. 4, Supplementary Tables S2 and S3). Pro acts as an osmolyte, ROS scavenger, and molecular chaperone for stabilizing protein structures^10,31^. Pro biosynthesis is dependent on the expression of the *P5CS* gene, and ABA stimulates Pro biosynthesis under drought-stress conditions^11,12^. In our study, ABA accumulation was associated with *TaP5CS* expression, which began on DT4 (Fig. 1F,I) before the metabolic response (proline accumulation) at DT6 (Supplementary Table S2). GABA also significantly accumulated on DT4, 8, and 10 (Supplementary Table S2). GABA accumulation has been associated with the carbon–nitrogen balance and ROS scavenging^32,33^. There is a possible correlation between GABA and Pro biosynthesis under drought-stress conditions.

The levels of BCAAs (Leu, Ile, and Val) increased significantly, starting from DT4, as the drought stress progressed. Previous studies suggested that BCAAs are an alternative source of energy in sugar-starved Arabidopsis^34^, and drought-stressed wheat^17^. Urano et al.^35^, in a dehydration experiment, reported high accumulations of BCAAs that were regulated at the transcript level by the BCAA biosynthesis enzyme, branched-chain aminotransferase (*BCAT2*). Thus, we concluded that the high BCAA accumulation level is an adaptive response to drought stress.

His biosynthesis is tightly linked to nucleotide metabolism through 5-phosphoribosyl-1-pyrophosphate^36^, which is required for the de novo biosynthesis and salvaging of nucleotides, as well as for plant growth and biomass accumulation^37^. In recent studies, Das et al.^38^ and Michaletti et al.^39^ reported that drought stress stimulates the upregulation of major purine bases as a first step in activating nucleic acid protective mechanisms. Purine–His cross-pathway regulation has been reported in *Saccharomyces cerevisiae*^40^. In the present study, concomitant His and nucleoside accumulations were observed starting from DT6 (Fig. 6 and Supplementary Table S2). Thus, His accumulation may be correlated with the biosynthesis and protection of nucleosides under drought-stress conditions. Although His biosynthesis is an energy-demanding process, consuming 41 ATP molecules per His molecule synthesized^41^, plant cells may preferentially synthesize His as a protectant of purine nucleosides under severe drought-stress conditions.

Purine metabolism is the fundamental route for nitrogen recycling and remobilization in non-leguminous plants^42^. Allantoin, a nitrogen-rich intermediate of purine catabolism, stimulates ABA production and jasmonic acid homeostasis in Arabidopsis under stress conditions^42,43^, indicating that purine metabolism plays dual roles in plants during stress. In the present study, pyrimidine metabolites, such as thymidine, uridine, and cytidine, and the purine metabolite allantoin accumulated only at DT10 (Table 2 and Supplementary Table S2), suggesting their involvement in severe drought responses. Allantoin increased by 120-fold, indicating an increase in nitrogen recycling, which is a survival mechanism under severe drought-stress conditions. At the same time, ABA increased by 23.8-fold (far more than the 9.6-fold at DT6), suggesting allantoin-stimulated ABA production, as previously reported^42,43^. We, therefore, concluded that nucleoside metabolism, especially allantoin accumulation, was not only involved in nitrogen recycling but also in the upregulation of ABA biosynthesis in response to severe drought stress.

Among the condition-specific compounds observed in this study, ACC was downregulated at DT4, 6, and 8, but upregulated at DT10 (13-fold change). ACC is the precursor of the phytohormone ethylene, and it also functions as a signal itself, independent from ethylene^44^. The accumulation pattern of ACC is largely associated with ABA, which increased by 23.8-fold at DT10 (Supplementary Table S2). Exogenous ABA applications accelerate the ageing processes in rice and maize^45–47^. In particular, ABA reduces the chlorophyll content in barley^48^. The rice *NAC2* gene, which is involved in ABA biosynthesis, has been reported to activate chlorophyll degradation genes, thereby accelerating ageing^49,50^. ACC upregulation at DT10 indicates an increase in ethylene signalling, which is correlated with accelerated ageing in response to severe drought-stress conditions. Thus, ABA may have stimulated senescence in wheat by cooperating with ethylene signalling in response to the severe drought stress. Interestingly, Asn, which accumulates in ageing leaves^51,52^, also accumulated under severe drought conditions (DT8 and 10), suggesting drought-induced senescence. In a recent study, metabolites belonging to the aspartate pathway (including Asn, Ser, and Met) were reported as biomarkers for yield gap-based drought tolerance, accurately predicting more than 94% of drought tolerance in wheat^4^. However, in our study, only methionine (among the aspartate pathway metabolites) made a large contribution to the drought response (Fig. 2A, Supplementary Table 3) and may be effective as a biomarker. The discrepancies among the results may be caused by differences in experimental conditions.

Aromatic amino acids (Phe, Trp, and Tyr) are synthesized through the shikimate pathway and are precursors to a wide range of secondary metabolites, such as terpenoids, auxins, glycosides, and lignin intermediates^53^. In free form, aromatic amino acids are targets of oxidation and have protective functions against ROS^54^. In our study, aromatic amino acids significantly accumulated under drought-stress conditions, starting from DT4 (Fig. 6 and Supplementary Table S2). This early accumulation may have contributed to the ROS scavenging capacity of the plant to prevent oxidative stress during the early stages of drought. Consequently, there was no significant ROS accumulation during the early drought stages. Significant ROS accumulations occur only when the plant scavenging capacity is overwhelmed by stress^55^. Therefore, we conclude that aromatic amino acids may have played a protective role against early drought-induced oxidative stress. Similarly, serotonin, a Trp-derived metabolite, significantly accumulated only at DT10 and may be involved in severe drought responses. In a recent report, serotonin was identified as a stress defense molecule that delays senescence in rice^56^. Hence, Trp metabolism was activated only at DT10 in response to drought-induced senescence and may be involved in anti-senescence activities. Interestingly, shikimic and ferulic acids decreased under drought-stress conditions (Fig. 7, Supplementary Fig. 2). Shikimic acid is the precursor of aromatic amino acids in the shikimate pathway, while ferulic acid is formed downstream of the shikimate pathway, starting with Phe and Tyr. The decrease in shikimic acid (upstream) and ferulic acid (downstream) suggest that the shikimate pathway was not responsible for the aromatic amino acid accumulations. We, therefore, conclude that the aromatic amino acid accumulations may have resulted from protein degradation under drought-stress conditions. These findings corroborate a recent report in which amino acid accumulations resulted from protein degradation in drought-stressed Arabidopsis^57^.

In summary, our findings indicate that the physiological phase-shift point of wheat under progressive drought stress is near DT6 (SWP = about − 400 kPa) . In addition, we have identified metabolites that play significant roles and are potential biomarkers for drought-stress responses. The condition-related specificities of these metabolites suggest a disruption in their respective pathways or relevant protein degradation induced by specific drought levels. However, these findings require validation, which can be achieved using a variety of genetic resources including drought-tolerant and drought-sensitive wheat lines^58,59^, to establish the applicability of these biomarkers in diverse genotypes. Interestingly, a highly diverse population, known as multiple synthetic derivative lines, has been developed by making wild introgressions using ‘Norin 61’ as a background genotype^60^. With the soon-to-be-released complete genomic sequence of ‘Norin 61’ (10 + genome project, www.10wheatgenomes.com), the future of wheat breeding using the multiple synthetic derivative lines seems promising, and this study will serve as a reference guide. Thus, this study has extended our knowledge of the metabolic and physiological dynamics in wheat in response to progressive drought stress. In the future, high-throughput analyses and validations of these findings will allow them to serve as effective tools in drought-tolerance breeding.

## Methods

### Plant material and growth condition

A standard Japanese spring wheat cultivar, ‘Norin 61’, was used for this study. ‘Norin 61’ is a representative wheat genome (10 + genome project, www.10wheatgenomes.com). Seeds were stratified at 4 °C for 7 d, hardened at room temperature for 24 h, and then transferred to pots (ϕ 7.5 cm × D 6.5 cm) containing commercial garden soil and maintained in a greenhouse for 30 d. The 30-d-old seedlings (4th-leaf-stage) were carefully transplanted into another soil medium prepared by watering dry commercial mixed soil (Oishii yasai wo sodateru tuchi, CAINZ, Japan) to field capacity with water containing 10 mL/L of liquid fertilizer (N P K 6. 10. 5, HYPONex, Japan). The medium was placed in rectangular pots (internal dimension = L 30 cm × W 18 cm × H 18 cm), with each pot containing three plants. A total of 16 pots containing 30-d-old plants were transferred to two climatic growth chambers (Espec, Japan). The growth chambers were maintained at 23 °C (14-h light)/19 °C (10-h dark), with relative humidity levels of 50% (light)/60% (dark), and a photosynthetic photon flux density of 900 µmol s^−1^ m^−2^. To avoid spatial heterogeneity, turntables within the growth chambers ensured constant changes in plant positions (Supplementary Fig. S1). The soil volumetric water content (SWC) was measured using moisture sensors (Em5b, Decagon devices, WA, USA), and the SWP was determined using the conversion equation^9^:

1 $${SWP=1606.8\times e}^{-17.69*SWC}$$

### Experimental design, drought treatment and sampling

The experiment was set up in a completely randomized design with the 16 pots being randomly assigned and subjected to either well-watered or progressive drought conditions. That is, each chamber had eight pots: four control and four drought pots. Progressive drought was initiated by withholding water at Zadok’s stage 65 (halfway into flowering^61^). Control pots were maintained at SWP − 15 kPa. Flag leaf samples were collected at d 0 (before the drought treatment, D0), 2, 4, 6, 8, and 10 after withholding water (DT2, 4, 6, 8, and 10, respectively). Four plants were sampled from each condition, with four flag leaf samples taken from each plant. Sampling was performed between 11:00 am and 12:00 noon (~ 5 h into photoperiod) to account for diurnal fluctuations. All the samples were snap-frozen with liquid nitrogen, pulverized with MULTI-BEADS SHOCKER (Yasui Kikai, Japan), and stored at − 80 °C.

### Canopy temperature measurement

Leaf thermal images were taken with an infrared camera (R500EX-Pro, NIPPON AVIONICS, Tokyo, Japan) on each sampling day just before flag leaf samples were collected. The images were taken laterally to minimize background effect due to the scanty nature of the plant canopy under severe drought stress. The thermal images were analysed using manufacturer’s software (NS9500LT Version 2.7A, NIPPON AVIONICS). Twenty data points were randomly selected per image, and the average value was recorded as the CT. The CTD was determined by subtracting CT from ambient temperature.

### Reactive oxygen species quantification

To estimate the extent of drought-induced oxidative stress, the ROS content was measured using a fluorogenic probe, 2,7-dichlorofluorescein (Cell Biolabs, San Diego, CA, USA) as described in Narayanan et al*.*^62^. Briefly, 50 mg of pulverized flag leaf samples were suspended in 1 ml of 1 × phosphate-buffered saline (pH 7.4) and centrifuged at 10,000 × *g* for 5 min. The supernatant (50 µL) was transferred to a black 96-well microplate and incubated for 5 min with a catalyst at room temperature. A freshly prepared dichlorodihydrofluorescein solution (100 µL) was added to each well and incubated for 45 min in the dark. After incubation, fluorescence from each well was read at 485 nm (excitation)/530 nm (emission) wavelengths using a microplate reader (SH-9000, Corona Electronic, Ibaraki, Japan). The amount of ROS was normalized using the sample fresh weight.

### Carbon isotope analysis

The ^13^C composition of flag leaves was analysed using an Elemental Analyser interfaced with a continuous-flow isotope ratio mass spectrometer (EA/IRMS; Thermo Fisher Scientific). Dried, pulverized flag leaf samples (1 mg) were filled into tin capsules (5 × 9 mm, LUDI Swiss) and placed in a combustion oven using an automatic sampler. Each sample was measured against standard CO_2_ calibrated with an isotope standard. The accuracy of calibration was ± 0.066‰ SD. Finally, the ^13^C composition was calculated as

2 $${\delta }^{13}C=\left[\left(\frac{Rsample}{Rstandard}\right)-1\right]\times \mathrm{1,000},$$

where R is the ^13^C/^12^C isotope ratios of samples and standards.

### Metabolite analysis: sample preparation and quantification

In total, 50 mg of each pulverized flag leaf sample was freeze-dried and stored in a desiccator at room temperature for metabolite analysis. Later, 4 mg of each freeze-dried sample was treated with 500 µL of 50% methanol and centrifuged at 15,000 × *g* at 4 °C for 5 min. Then, to separate polar and non-polar metabolites, 450 µL of the supernatant was carefully mixed with an equal volume of chloroform, vortexed and centrifuged (15,000 × *g* at 4 °C for 5 min). In total, 400 µL of the resultant supernatant was filtered through a membrane (Amicon Ultra-0.5 mL, 3 kDa cutoff, Millipore, Billerica, MA, USA) and centrifuged (15,000 × *g* at 4 °C for 30 min). MiliQ water (400 µL) was added to the filter and centrifuged (15,000 × *g* at 4 °C for 30 min). The filtrate was then dried in a SpeedVac concentrator (Thermo Fisher Scientific, Waltham, MA, USA) at 45 °C for 6 h. The concentrated dry sample was resuspended in 50% methanol (200 µL). A 50-µL aliquot was then transferred into another tube containing 450 µL of 50% methanol to form a tenfold dilution. The resulting 500 µL solution was used for LC–MS metabolite quantification. In total, 94 metabolites were quantified using a triple quadrupole LC–MS/MS system (Agilent 6420, CA, USA), with a Discovery HS-F5 column (2.1 × 250 mm, 5 μm, Sigma-Aldrich, PA, USA). The metabolites were identified by MRM analysis. Product ions used to characterize each metabolite are shown in Supplementary Table S4. The levels of metabolites in each leaf sample were normalized using sample dry weight. A quality control reference was established using metabolite standard mixtures of different concentrations (0, 0.4, 2 and 10 ppm). Compounds having similar molecular masses or retention times were not included in the same mixture. The mobile phase consisted of 0.1% formic acid and acetonitrile as A and B solutions, respectively. A gradient flow with four A:B ratios was applied: (1) 100% A:0% B for 2 min, (2) 72% A:25% B for 8 min, (3) 65% A:35% B for 4 min, and (4) 5% A:95% B for 3 min. All the solvents and reagents used were of LC–MS grade.

### Quantitative reverse transcription PCR (qRT-PCR) analysis

#### RNA extraction

Total RNA was isolated from flag leaves using RNeasy Plant Mini Kit (74904; Qiagen, Germany) according to the manufacturer’s protocol. A total of 500 ng total RNA was reverse transcribed using ReverTra Ace qPCR RT Master Mix with gDNA remover according to the manufacturer’s manual (Toyobo, Japan).

#### Primer design

Sequence homologs of three ABA-responsive genes were queried against the hexaploid wheat sequences obtained from the International Wheat Sequencing Consortium using the Phytozome database (https://www.phytozome.net). The genes are (1) an ABA-signalling regulator, *TaPP2C6*; (2) a late embryogenesis abundance protein, *TaLEA*^9^; and (3) a Pro biosynthetic gene, *TaP5CS*^24^. The sequences with the highest homology levels to the hexaploid wheat genome were selected. Primer sequences were designed using NCBI PrimerBlast (https://www.ncbi.nlm.nih.gov/tools/primer-blast/).

#### qRT-PCR for ABA-responsive genes

qRT-PCR was performed on a StepOnePlus Real Time PCR system (Life Technologies) using KOD SYBR qPCR Mix (Toyobo), and the gene-specific primer sets are shown in Supplementary Table S1. The PCR program consisted of an initial temperature of 98 °C for 2 min, followed by 40 cycles of 98 °C for 10 s, 60 °C for 10 s and 68 °C for 30 s. A melting curve was constructed by increasing the temperature from 68 to 99 °C at a rate of 0.05 °C s^−1^. To calculate the copy number, a standard curve was generated for the pMD20 plasmid containing the target DNA sequence. Four biological replicates were performed, and *TaActin* was used as an internal standard for normalization.

### Statistical analyses

ANOVA, Student’s *t*-tests, and *Z*-transformation of metabolic and physiological data were conducted using Microsoft Excel 2019. A principal component analysis (PCA) and graphical representations were made using the R program, version 3.5.2^63^. The metabolite pathway and network analysis were conducted using Kyoto Encyclopedia of Genes and Genomes (KEGG; https://www.genome.jp/kegg/) and the literature. The cluster analysis was conducted using the Mass Profiler Professional software (MPP version 2.5, Agilent Technologies, CA, USA).

## Supplementary information

Supplementary figures.

Supplementary tables.
