# Supplementary material for: Metabolic and physiological responses to progressive drought stress in bread wheat
Source: Sci Rep. 2020 Oct 14;10:17189. doi: 10.1038/s41598-020-74303-6 (PMC7560863; doi:10.1038/s41598-020-74303-6)
Supplement: Supplementary file 1 — Supplementary figures. [file 41598_2020_74303_MOESM1_ESM.pdf]

Title:

Metabolic and physiological responses to progressive drought stress in bread wheat

Author:

Michael Itam, Ryosuke Mega, Shota Tadano, Mostafa Abdelrahman, Sachiko Matsunaga, Yuji Yamasaki, Kinya Akashi and Hisashi Tsujimoto

Fig.S1

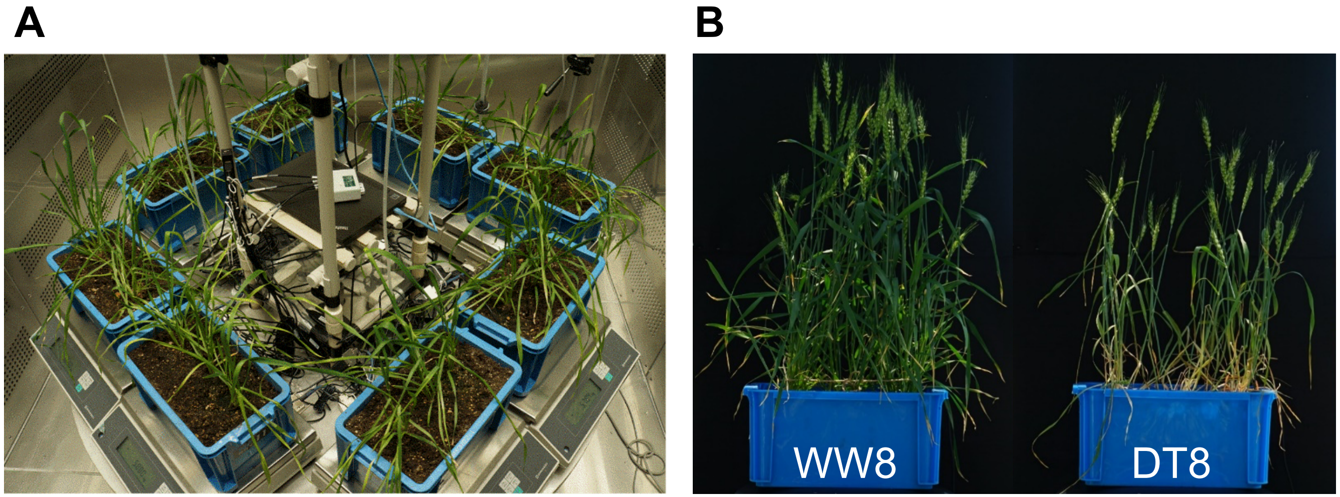

Fig.S2

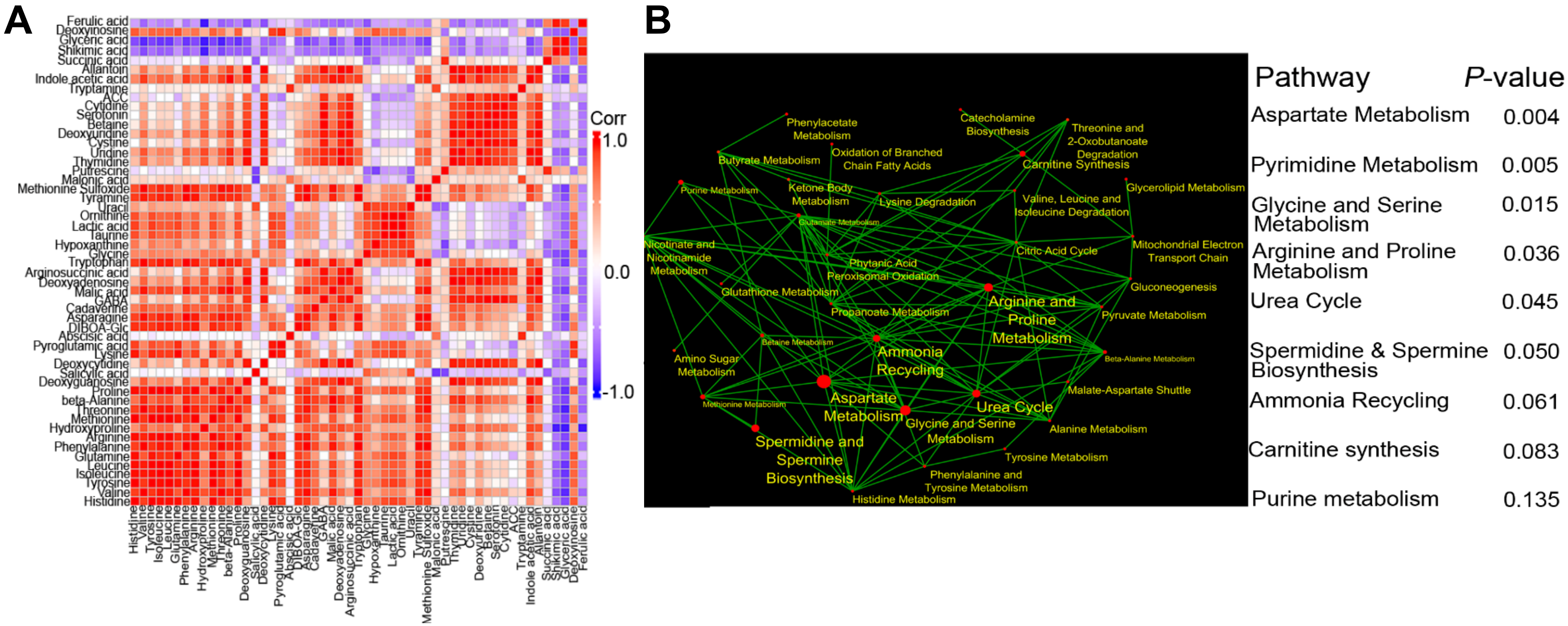

Pathway	*P*-value
Aspartate Metabolism	0.004
Pyrimidine Metabolism	0.005
Glycine and Serine Metabolism	0.015
Arginine and Proline Metabolism	0.036
Urea Cycle	0.045
Spermidine & Spermine Biosynthesis	0.050
Ammonia Recycling	0.061
Carnitine synthesis	0.083
Purine metabolism	0.135
